# Motor Performance Assessment in Parkinson’s Disease: Association between Objective In-Clinic, Objective In-Home, and Subjective/Semi-Objective Measures

**DOI:** 10.1371/journal.pone.0124763

**Published:** 2015-04-24

**Authors:** Nima Toosizadeh, Jane Mohler, Hong Lei, Saman Parvaneh, Scott Sherman, Bijan Najafi

**Affiliations:** 1 Interdisciplinary Consortium on Advanced Motion Performance (iCAMP) and Southern Arizona Limb Salvage Alliance (SALSA), Department of Surgery, College of Medicine, University of Arizona, Tucson, United States of America; 2 Arizona Center on Aging, University of Arizona, Tucson, United States of America; 3 Department of Neurology, College of Medicine, University of Arizona, Tucson, United States of America; University of Tuebingen, GERMANY

## Abstract

Advances in wearable technology allow for the objective assessment of motor performance in both in-home and in-clinic environments and were used to explore motor impairments in Parkinson’s disease (PD). The aims of this study were to: 1) assess differences between in-clinic and in-home gait speed, and sit-to-stand and stand-to-sit duration in PD patients (in comparison with healthy controls); and 2) determine the objective physical activity measures, including gait, postural balance, instrumented Timed-up-and-go (iTUG), and in-home spontaneous physical activity (SPA), with the highest correlation with subjective/semi-objective measures, including health survey, fall history (fallers vs. non-fallers), fear of falling, pain, Unified Parkinson's Disease Rating Scale, and PD stage (Hoehn and Yahr). Objective assessments of motor performance were made by measuring physical activities in the same sample of PD patients (n = 15, Age: 71.2±6.3 years) and age-matched healthy controls (n = 35, Age: 71.9±3.8 years). The association between in-clinic and in-home parameters, and between objective parameters and subjective/semi-objective evaluations in the PD group was assessed using linear regression-analysis of variance models and reported as Pearson correlations (*R*). Both in-home SPA and in-clinic assessments demonstrated strong discriminatory power in detecting impaired motor function in PD. However, mean effect size (0.94±0.37) for in-home measures was smaller compared to in-clinic assessments (1.30±0.34) for parameters that were significantly different between PD and healthy groups. No significant correlation was observed between identical in-clinic and in-home parameters in the PD group (*R* = 0.10–0.25; *p*>0.40), while the healthy showed stronger correlation in gait speed, sit-to-stand duration, and stand-to-sit duration (*R* = 0.36–0.56; *p*<0.03). This suggests a better correlation between supervised and unsupervised motor function assessments in healthy controls compared to PD group. In the PD group, parameters related to velocity and range-of-motion of lower extremity within gait assessment (*R* = 0.58–0.84), and turning duration and velocity within iTUG test (*R* = 0.62–0.77) demonstrated strong correlations with PD stage (*p*<0.01).

## Introduction

Parkinson’s disease (PD) is the second most common neurodegenerative disease after Alzheimer’s disease, with over 60,000 new diagnoses each year in the United States alone [1]. Motor impairment due to basal ganglia dysfunction causes gait and balance deterioration, increased risk of falling, and an overall reduced quality of life in people living with PD [2]. Several objective outcomes of motor impairment have been previously introduced to explore differences between healthy controls and PD patients, as well as pre- and post-treatment changes in PD conditions, including gait analysis [3], postural balance performance [4,5], Timed-up-and-go (TUG) [6], and spontaneous physical activity (SPA) [7,8].

Common objective motor assessments use a gait protocol inclusive of spatio-temporal parameters such as gait speed, cadence, stride length, and gait asymmetry [3,9]. Postural balance is assessed using magnitude and direction of body sway and implemented strategy for maintaining balance (i.e., ankle-strategy/hip-strategy or dependency on sensory feedback) [4,5]. Further, Horak et al. have developed a metric for mobility assessment in PD, which includes: postural stability in stance, postural responses, gait initiation, gait (temporal-spatial lower and upper body coordination and dynamic equilibrium), postural transitions, and freezing of gait [10]. Traditionally, the TUG test was the sole temporal measure used to assess motor performance. More recently, a new sensor-based instrumented TUG (iTUG) method, which includes spatio-temporal parameters during turning, standing, and sitting, demonstrated more sensitivity in differentiating between PD patients and healthy controls [6]. Finally, by using wearable sensors, the differences in SPA parameters, such as gait speed, transitions from sit-to-stand and stand-to-sit, and the number and duration of walking episodes have been quantified between PD patients and healthy controls [7,8].

Although previous studies have demonstrated motor impairments in PD patients, it is unclear whether unsupervised in-home motor function assessment is as sensitive as supervised in-clinic measures when used to identify motor impairment due to PD. Furthermore, performing several objective tests to assess longitudinal changes in motor performance could be difficult in terms of patient burden and cost; therefore, it is important to identify those physical tasks which are most sensitive to changes in the PD stage and symptom profile. The goal of the current study was to assess gait, postural balance, iTUG, and SPA in a sample of PD patients and healthy controls in order to assess correlations between in-clinic and in-home physical activity parameters (i.e., gait speed, sit-to-stand duration, and stand-to-sit duration). We also explored the association between the severity of PD and motor impairments using the above objective measures, correlating objective parameters and subjective (e.g., fear of falling and history of falls based on fallers and non-fallers categories) and semi-objective evaluations (e.g., Unified Parkinson's Disease Rating Scale (UPDRS) and the Hoehn and Yahr (H&Y) disease stage).

## Methods

### Participants

Fifteen aging adults over 55 years with idiopathic PD, diagnosed by movement disorder specialists based on the UK Brain Bank criteria, were recruited from the University of Arizona Neurology Clinic. Participants were excluded if they were diagnosed with any type of neurological disorder other than PD, had received previous PD surgical treatments such as deep brain stimulation, or progressed to stage five PD based on H&Y staging. A sample of 35 healthy, non-frail (measured by Fried Frailty index [11]) adult controls, over 55 years of age, were recruited from the University of Arizona Geriatric clinic. Elders with major mobility disorder (unable to walk a distance of 20 meters without walking assistance) or balance impairments (unable to perform balance trials as explained below) were excluded. The study was approved by the University of Arizona Institutional Review Board, and written informed consent according to the principles expressed in the Declaration of Helsinki [12] was obtained from all subjects before participation.

### Objective assessment

Objective assessments included: 1) gait; 2) postural balance; 3) iTUG; and 4) SPA. Several sensor-based outcomes were extracted including: spatio-temporal, smoothness, and asymmetry parameters of gait; body sway and balance controlling strategy parameters for postural balance; trunk velocity and range of motion during the iTUG test; and walking, sitting, standing, and lying duration, number and speed of gait and postural transition for the SPA monitoring (see Tables 1 and 2 for the detailed list of parameters). Of note, the current study presented only the biomechanical and physiological definitions of the parameters; readers are referred to previous studies for approaches to calculations (Tables 1 and 2). Of all the parameters that could be extracted, the more commonly used and physiologically meaningful ones were assessed in this study.

**Table 1:** Objective parameter definitions from gait and postural balance tests.

Gait	Definition	Reference
Steps to steady state	Number of steps before the steady state walking *	[13]
Distance to steady state	Distance travelled before achieving steady state walking *	[13]
Gait speed	Steady state gait speed *	[14]
Stride length	Distance travelled by the same limb between two successive heel contacts during the steady state walking *	[14]
Gait cycle time	Time interval starts when one foot makes contact with the ground and ends when that same foot contacts the ground again during the steady state walking *	[14]
Initial double support	First foot strike to opposite foot’s toe-off duration as a percentage of gait cycle time during the steady state walking *	[14]
Terminal double support	Duration of walking begins with the opposite foot strike until toe off as a percentage of gait cycle time during the steady state walking *	[14]
Double support	Duration of the initial and terminal double support as a percentage of gait cycle time during the steady state walking *	[14]
Right or left knee angle	Range of right or left knee angular movement in the sagittal plane during the steady state walking *	[14]
Speed variability	Coefficient of variation (SD divided by the mean) of gait speed among gait cycles during the steady state walking *	[14]
Mid-swing velocity	Mean value of shank angular velocity peaks during each swing for strides during the steady state walking *	[14]
Gait smoothness	A measure of movement quality and the step-to-step symmetry within a stride, measured by harmonic ratio of vertical trunk movement	[15]
Knee asymmetry	Percentage difference in the range of angular movement between right and left knee	-
**Postural Balance**		
Ankle sway	Product of range of ankle rotations in the anterior-posterior and medial-lateral direction	[16]
Hip sway	Product of range of hip rotations in the anterior-posterior and medial-lateral direction	[16]
Ankle/hip sway	Ankle sway over hip sway	[16]
COG_AP_	Range of COG sway in the anterior-posterior direction	[17]
COG_ML_	Range of COG sway in the medial-lateral direction	[17]
COG	Product of COG_AP_ and COG_ML_	[17]
COG_ML/AP_	COG_ML_ over COG_AP_	[17]
RCI_AP_ or RCI_ML_	Representing correlation between hip and ankle motions in the anterior-posterior or medial-lateral direction	[17]
OLslope_ML/AP_	Representing the amount of medial-lateral over anterior-posterior sway during the open-loop control (i.e., controlling mechanism that only involves postural muscles not sensory feedback)	[18]
OLΔt_AP_ or OLΔt_ML_ or OLΔt	Represents the maximum time-interval, in which, sensory feedback is added to the open-loop control(in anterior-posterior direction, medial-lateral direction, or overall)	[18]

A reference for calculation procedure is presented for each parameter.

COG: center of gravity

AP: anterior-posterior

ML: medial-lateral

RCI: reciprocal compensatory index

OL: open-loop

* Steady-state walking was the first stride of the group of six strides with an SD below the median SD of the all analyzed strides ±6% [13].

**Table 2:** Objective parameter definitions from iTUG and SPA tests.

iTUG	Definition	Reference
S-St duration *	Required time to stand up from a chair	[6]
Walk duration	Required time to walk three meters forward and backward	[6]
Turn duration †	Required time for the first and second turn	[6]
St-S duration *	Required time to sit down on a chair	[6]
Total duration	Required time to perform the entire iTUG task	[6]
S-St%, walking%, first turn%, or second turn + St-S% * †	Duration of sit-to-stand, walking, first turn, or second turn + stand-to-sit as a percentage of the total iTUG duration	[6]
Turning, S-St, or St-S peak velocity * †	Maximum angular velocity of the trunk during turning, rising from a chair, or sitting on a chair	[6]
Turning, S-St, or St-S mean velocity * †	Mean angular velocity of the trunk during turning, rising from a chair, or sitting on a chair	[6]
S-St or St-S angular ROM * †	Range of trunk angle during rising from a chair or sitting on a chair	[6]
**SPA**		
Sitting%, standing%, walking%, or lying %	Percentage of sitting, standing, walking, or lying in one day monitoring	[19]
Walking episodes	Number of walking episodes in one day monitoring (more than three strides in five seconds)	[19]
Total steps	Total number of steps in one day monitoring	[19]
Max steps	Maximum number of steps in one episode of walking	[19]
Gait speed ‡	Mean gait speed in one day monitoring for walking episodes with duration of greater than five seconds and minimum three consecutive steps	[20]
S-St or St-S number *	Number of sit-to-stand or stand-to-sit in one day monitoring	[19]
S-St or St-S duration *	Mean duration of sit-to-stand or stand-to-sit in one day monitoring	[19]

A reference for calculation procedure is presented for each parameter.

iTUG: instrumented Timed-up-and-go

SPA: spontaneous physical activity

S-St: sit-to-stand

St-S: stand-to-sit

ROM: range of motion

* St-S and St-S transition were defined based on the change of trunk tilt in the sagittal plane both in the iTUG and SPA assessments.

†Turn transition was defined based on the change of trunk twisting angle in the iTUG assessment.

‡ Gait speed was computed using information from the detected step time and the amplitude of acceleration during each gait cycle.

1. Gait: To assess gait performance, participants performed two tests of normal gait (> 25 steps) under single task and dual task conditions. The dual-task condition for gait was used since previous research has demonstrated an exacerbated motor performance, specifically during gait, in PD patients while performing in a dual-task condition [21]. The dual-task measure of counting backwards by one was chosen here, since this task has been proven to be simpler and more appropriate for older adults [22].
2. Postural balance: Each participant then performed four 30-second trials of balance assessment; in each trial participants stood upright with their feet as close together as possible without touching each other and with their arms crossed to minimize the effect of arm movements on center of gravity (COG) displacements. In the first two trials, participants were instructed to keep their eyes open, with no visual target specified. In the third and fourth trials participants closed their eyes [16].
3. iTUG: The iTUG task was performed by asking the participants to stand up from a standard chair without using their arms, walk three meters, turn, and walk back and sit down on the same chair.
  Three-dimensional acceleration and angular velocity of shanks, thighs and the trunk were measured using five wearable sensors each included a triaxial accelerometer and a triaxial gyroscope (LEGSys and BalanSens—BioSensics, Boston, MA) to derive gait, postural balance, and iTUG outcome measures following procedures identical to those explained in earlier studies (see Tables 1 and 2). Sensors were attached to the shank above the ankle, to the thigh above the knee, and to the lower back in the lumbar region. All in-clinic outcomes were assessed in an “off-medication state” (i.e., >12 hours after the last PD medication dose).
4. SPA: To measure SPA, a previously validated method involving a wearable technology was used (PAMSys, BioSensics, Boston, MA) [7,8]. Briefly, this method utilized a tri-axial accelerometer placed inside a shirt in a pocket close to the sternum to estimate the duration and number of various spontaneous activities at home as listed in Table 2. Parameters were measured for one day (24 hours consecutively during a weekday).

### Subjective and semi-objective evaluations

Subjective/semi-objective measures for the PD group included the SF-12 health survey [23], fall status, Short Falls Efficacy Scale-International (Short FES-I) [24], the visual analog scale (VAS) for pain [25], UPDRS (parts I, II, and III) [26], and H&Y staging (disease stage) [27]. SF-12 health survey was used to assess generic health status based on physical and mental components. Fall history was recorded to categorize participants into faller and non-faller groups; the faller category consisted of participants with one or more falls during the past year. Short FES-I was used to assess fear of falling while doing several daily activities to estimate the contribution of fear of falling to functional decline among elderly people [28]. Since all objective and subjective/semi-objective measurements were performed in one visit, shorter validated versions of questionnaires were used for both SF-12 health survey (12 instead of 36-item) and Short FES-I (7 instead of 16-item) to minimize the burden on PD patients. VAS pain assessment was performed, since pain is commonly attributed to PD [29,30]. Furthermore, UPDRS was used as a common practice for PD to rate patient’s disability based on: 1) mentation, behavior and mood (part I); 2) activities of daily living (part II); and 3) motor examination (part III). Both UPDRS and H&Y staging were assessed by two examiners (in the clinic and using recorded video).

### Statistical Analysis

For the first aim of the study, correlations between in-clinic and in-home parameters were assessed using linear regression-analysis of variance (ANOVA) models for gait speed, sit-to-stand duration, and stand-to-sit duration (see Table 2 for definitions) and reported as Pearson correlations (*R*). Cut-offs of 0.01–0.19: negligible, 0.20–0.29: weak, 0.30–0.39: moderate, 0.40–0.69: strong, and 0.70–1.00: very strong were selected for correlations [31]. Although several parameters were obtained from in-clinic measurements, comparisons were made only between those parameters that were also measureable within in-home assessments. For the second aim of the study, first a comparison between healthy controls and the PD group for objective parameters was performed using unpaired *t*-test (or Mann–Whitney U test for non-parametric samples) and Cohen’s effect size was calculated. Then, for objective parameters that were significantly different between the PD and control groups, the association between objective parameters and subjective/semi-objective evaluations in the PD group was assessed using linear regression-ANOVA models and reported as Pearson correlations. ANOVA tests were used to assess differences in objective physical activity measurements between fallers and non-fallers in the PD group. A summary of results is presented as mean (standard deviation—SD). All analyses were done using JMP (Version 10, SAS Institute Inc., Cary, NC), and statistical significance was concluded when p ≤ 0.01. The level of 0.01 was selected for significance to more conservatively determine the difference in parameters between the PD and healthy control groups.

## Results

### Participants

Mean (SD) age and body mass index (BMI) of PD participants were 71.2 (6.3) years and 27.5 (6.5) kg/m^2^, respectively. Corresponding values for the healthy controls were 71.9 (3.8) years and 25.7 (4.4) kg/m^2^. Based on H&Y staging, two (13%) PD participant were in stage 1, two (13%) were in stage 2, one (7%) was in stage 2.5, seven (47%) were in stage 3, and three (20%) were in stage 4. Other sociodemographic information is reported in Table 3.

**Table 3:** Mean (SD or percentage) values of participant sociodemographic information.

	PD	Healthy	95% CI		*p*-value
Number, % of total	15 (30%)	35 (70%)	-	-	-
Male (% of the group)	8 (53%)	10 (29%)	0.1	1.4	0.07
Age, years (SD)	71.2 (6.3)	71.9 (3.8)	-5	3	0.71
Stature, cm (SD)	164.3 (10.9)	163.0 (5.7)	-5	7.5	0.52
Body mass, kg (SD)	74.9 (15.3)	68.3 (12.6)	-2.6	16	0.08
BMI, kg/m^2^ (SD)	27.5 (6.5)	25.7 (4.4)	-1	5	0.91
MMSE score (SD)	25.7 (5.6)	29.2 (1.1)	0.6	1.5	<.0001*
Fallers (% of the group)	7 (47%)	9 (26%)	-0.2	1.1	0.15
VAS, 0–10 (SD)	2.8 (3.1)	0.5 (1.0)	-1.7	-0.5	<.001*
SF-12, MCS (SD)	44.7 (11.0)	-	-	-	-
SF-12, PCS (SD)	38.1 (10.9)	-	-	-	-
Short FES-I, 7–28 (SD)	17.0 (6.0)	-	-	-	-
PD H&Y stage, 0-5(SD)	2.9 (0.9)	-	-	-	-
UPDRS I, 0–16 (SD)UPDRS II, 0–52 (SD)UPDRS III, 0–104 (SD)	4.6 (3.2)	-	-	-	-
	17.7 (8.4)	-	-	-	-
	34.8 (13.9)	-	-	-	-
Disease duration, years (SD)	5.9 (5.3)	-	-	-	-
Total LEDD (SD)	517 (380)	-	-	-	-
Dopamine agonist LEDD (SD)	56 (90)	-	-	-	-

The symbol * indicates a significant difference using *t*-test or U test (0.01 was used as the significance level).

PD: Parkinson’s disease

CI: confidence intervals

BMI: body mass index

MMSE: Mini-Mental State Examination [32]

MCS: mental health composite scale

PCS: physical health composite scale

FES-I: Falls Efficacy Scale-International

VAS: visual analog scale

H&Y stage: Hoehn and Yahr stage

UPDRS: Unified Parkinson’s Disease Rating Scale

LEDD: regular levodopa dose x 1 + levodopa continuous release dose x 0.75 + (regular levodopa dose + continuous release levodopa dose x 0.75) x 0.25 if taking tolcapone or entacapone) + pramipexole dose x 67 + ropinirole dose x 20 + rotigotine x 30 + pergolide dose x 100 + bromocriptine dose x 10 + cabergoline dose x 50 + amantadine dose x 0.5 + selegiline dose x 10 + resagiline dose x 100.

### Association between in-clinic and in-home physical activity parameters

Overall, no significant correlation was observed between identical in-clinic and in-home parameters in the PD group (*p* > 0.40); correlation coefficients (*R*) were 0.25, 0.13, and 0.10 for sit-to-stand duration, stand-to-sit duration, and gait speed, respectively. On the other hand, when comparing in-clinic and in-home outcome measures, the healthy control group showed stronger correlations in gait speed (*R* = 0.36; *p* = 0.03), sit-to-stand duration (*R* = 0.56; *p* < 0.01), and stand-to-sit duration (*R* = 0.47; *p* < 0.01).

### Association between objective parameters and subjective/semi-objective evaluations

Gait speed, knee angle during walking, mid-swing shank velocity, and gait smoothness were less, and gait cycle time was longer among PD patients compared to healthy controls in both normal habitual and dual-task conditions within gait assessments (Table 4). Comparing the balance behaviors between the PD and healthy control groups showed that hip sway and COG sway were smaller and range of medial-lateral over anterior-posterior sway was larger in the PD group (Table 4); balancing strategies were also different between the two groups (reciprocal compensatory index (RCI_AP_) and time interval of open-loop control (OLΔt)) both in eyes-open and eyes-closed conditions (see Table 1 for parameters definitions). Among iTUG parameters, turning duration and velocity, and trunk angle and angular velocity during sit-to-stand and stand-to-sit transitions had the largest effect size (Table 5). Finally, daily walking percentage, the number of steps during one day, and the duration of sit-to-stand and stand-to-sit from SPA differed between the PD and healthy control groups (Table 5). Among SPA parameters, those that associated with maximum number of steps during 24-hour monitoring demonstrated larger differences between the two groups.

**Table 4:** Differences in gait and postural balance parameters between PD and healthy control groups.

**Gait (Normal Speed)**	**PD**	**Healthy**	**95% CI**		***p*-value**	**Effect Size**
Steps to steady state	3.40 (1.76)	2.03 (0.82)	0.36	2.38	<.01*	1.00
Distance to steady state (m)	1.62 (1.21)	1.04 (0.80)	-0.13	1.29	0.21	0.20
Gait speed (m/s)	0.94 (0.30)	1.19 (0.13)	-0.42	-0.08	<.01*	1.09
Stride length (m)	1.11 (0.28)	1.27 (0.08)	-0.32	-0.01	0.04	0.80
Gait cycle time (s)	1.24 (0.15)	1.09 (0.10)	0.06	0.24	<.001*	1.18
Initial double support (%)	14.05 (2.96)	11.38 (2.31)	0.89	4.45	<.01*	1.00
Terminal double support (%)	13.53 (3.79)	11.11 (2.61)	0.18	4.66	0.04	0.74
Double support (%)	27.58 (6.20)	22.49 (4.18)	1.45	8.75	<.01*	1.00
Right Knee angle (deg)	43.37 (9.95)	56.48 (7.28)	-19.04	-7.19	<.001*	1.50
Left knee angle (deg)	40.42 (9.80)	56.54 (6.46)	-22.05	-10.53	<.0001*	2.00
Speed variability (%)	4.64 (2.78)	3.53 (1.83)	-0.53	2.75	0.20	0.47
Initial mid-swing (deg/s)	273.16 (83.25)	360.59 (32.50)	-134.50	-40.36	<.01*	1.38
Steady state mid-swing (deg/s)	279.89 (80.79)	360.47 (32.47)	-34.85	-126.31	<.01*	1.31
Initial gait smoothness	1.92 (0.37)	2.50 (0.45)	-0.32	-0.82	<.0001*	1.39
Steady state gait smoothness	2.13 (0.58)	2.91 (0.78)	-0.38	-1.18	<.001*	1.14
Knee asymmetry (%)	0.15 (0.09)	0.08 (0.06)	0.01	0.12	<.01*	0.92
**Postural Balance (eyes-open)**	**PD**	**Healthy**	**95% CI**		***p*-value**	**Effect Size**
Ankle sway (deg^2^)	3.51 (4.71)	4.34 (8.36)	-4.59	2.93	0.66	0.12
Hip sway (deg^2^)	2.04 (2.21)	3.85 (3.56)	-3.48	-0.13	0.01*	0.61
Ankle/hip sway	1.45 (0.49)	1.46 (3.32)	-1.18	1.15	0.98	0.00
COG_AP_ (cm)	0.63 (0.44)	1.19 (0.77)	-0.92	-0.22	<.001*	0.90
COG_ML_ (cm)	0.69 (0.45)	0.57 (0.33)	-0.15	0.39	0.21	0.30
COG (cm^2^)	0.55 (0.81)	0.80 (1.11)	-0.82	0.32	0.06	0.26
COG_ML/AP_	1.33 (0.68)	0.55 (0.28)	0.39	1.16	<.0001*	1.50
RCI_AP_	0.43 (0.18)	0.78 (0.17)	-0.45	-0.23	<.0001*	1.98
RCI_ML_	0.83 (0.07)	0.85 (0.19)	-0.09	0.06	0.76	0.14
OLslope_ML/AP_	2.34 (2.11)	0.65 (0.61)	0.51	2.87	<.001*	1.09
OLΔt_AP_ (s)	1.80 (0.80)	2.06 (0.88)	-77.53	26.27	0.34	0.31
OLΔt_ML_ (s)	2.54 (0.86)	2.32 (0.86)	-32.75	76.88	0.31	0.26
OLΔt (s)	1.32 (0.56)	2.13 (0.99)	-125.14	-35.81	<.01*	1.00

The symbol * indicates a significant difference using *t*-test or U test (0.01 was used as the significance level). Due to similarity between conditions, only normal habitual gait and the eyes-open condition for the balance test are presented. Mean (SD) are presented.

PD: Parkinson’s disease

CI: confidence interval

COG: center of gravity

AP: anterior-posterior

ML: medial-lateral

RCI: reciprocal compensatory index

OL: open-loop.

**Table 5:** Differences in iTUG and SPA parameters between PD and healthy control groups.

**iTUG**	**PD**	**Healthy**	**95% CI**		***p*-value**	**Effect Size**
S-St duration (s)	1.86 (9.41)	1.51 (5.46)	-0.19	0.90	0.23	0.05
Walk duration (s)	5.71 (3.78)	4.84 (1.02)	-1.24	2.99	0.99	0.31
Turn duration (s)	6.18 (4.06)	3.21 (4.62)	0.71	5.22	<.0001*	1.03
St-S duration (s)	2.05 (0.77)	2.19 (1.28)	-0.73	0.45	0.76	0.13
Total duration (s)	14.14 (6.07)	10.88 (1.80)	-0.13	6.67	0.06	0.74
S-St %	14.22 (5.74)	13.80 (3.91)	-2.97	3.81	0.91	0.09
Walking %	37.53 (10.90)	44.63 (6.89)	-13.47	-0.72	0.03	0.78
First turn %	21.11 (6.82)	14.54 (2.97)	2.69	10.44	<.01*	1.25
Second turn + St-S %	27.14 (10.78)	27.02 (6.84)	-6.19	6.42	0.35	0.01
Turning peak velocity (deg/s)	153.41 (46.70)	221.17 (31.01)	-95.24	-40.29	<.0001*	1.71
S-St peak velocity (deg/s)	117.10 (80.61)	125.49 (33.40)	-54.09	37.30	0.15	0.14
St-S peak velocity (deg/s)	102.02 (47.05)	110.33 (41.04)	-37.21	20.59	0.49	0.18
Turning mean velocity (deg/s)	66.91 (27.63)	105.12 (13.42)	-54.01	-22.40	<.0001*	1.76
S-St mean velocity (deg/s)	27.02 (14.33)	42.94 (11.82)	-24.62	-7.21	<.001*	1.21
St-S mean velocity (deg/s)	24.30 (10.65)	37.56 (9.43)	-19.82	-6.69	<.001*	1.32
S-St ROM (deg)	26.92 (12.03)	39.65 (11.21)	-20.22	-5.24	<.01*	1.09
St-S ROM (deg)	28.27 (11.73)	43.15 (14.07)	-22.72	-7.03	<.001*	1.15
**SPA**	**PD**	**Healthy**	**95% CI**		***p*-value**	**Effect Size**
Sitting %	44.11 (16.39)	43.93 (16.08)	-10.16	10.51	0.97	0.01
Standing %	14.40 (7.79)	16.96 (6.18)	-7.26	2.14	0.27	0.36
Walking %	6.02 (3.83)	9.18 (4.12)	-5.63	-0.68	0.01*	0.79
Lying %	35.36 (22.01)	29.81 (16.30)	-7.59	18.69	0.59	0.27
Walking episodes	381 (205)	524 (215)	-274.01	-10.39	0.04	0.44
Total steps	4099 (2673)	6391 (3217)	-4082	-502	0.01*	1.35
Max steps	189 (290)	346 (378)	-360	40	<.01*	0.47
Gait speed (m/s)	0.66 (0.11)	0.62 (0.04)	-0.02	0.10	0.18	0.48
Sit-to-stand number	90.27 (37.94)	120.97 (45.66)	-56.12	-5.29	0.04	0.73
Sit-to-stand duration (s)	4.63 (1.07)	3.86 (0.72)	0.14	1.40	<.01*	0.85
Stand-to-sit number	90.27 (37.94)	120.97 (45.66)	-56.12	-5.29	0.04	0.73
Stand-to-sit duration (s)	4.75 (0.93)	3.84 (0.74)	0.35	1.47	<.01*	1.09

The symbol * indicates a significant difference using *t*-test or U test (0.01 was used as the significance level). Mean (SD) are presented.

PD: Parkinson’s disease

iTUG: instrumented Timed-up-and-go

SPA: spontaneous daily physical activity

CI: confidence intervals

S-St: sit-to-stand

St-S: stand-to-sit

ROM: range of motion.

Assessment of the above parameters that were different between the PD and healthy control groups showed that, in the PD group, gait speed, stride length, knee angle during walking, and mid-swing shank velocity within gait assessment (*R* = (0.58–0.84)), and turning duration and velocity, and trunk angle and angular velocity during sit-to-stand within iTUG test (*R* = (0.62–0.77)) demonstrated strong or very strong correlations with the SF-12 health survey, short-FES-I, UPDRS sub-scores, and the disease stage (Table 6). Among postural balance parameters, only the time interval of open-loop control (OLΔt)) demonstrated significant correlation with UPDRS I, both in eyes-open and eyes-closed conditions (*R* = (0.46–0.66)). None of the SPA parameters were significantly associated with any of the subjective/semi-objective measures (*p* > 0.05). Furthermore, no significant correlation was determined between VAS score and any of the objective parameters in our sample. None of the postural balance and SPA parameters were significantly different between the faller and non-faller groups (*p* > 0.02). Alternatively, turning peak velocity within the iTUG test was significantly smaller among fallers with 88% slower turning velocity observed compared to non-fallers (*p* = 0.005). Within the gait parameters, stride length was smaller in the faller group in both normal (95%) and dual-task (85%) conditions; however, based on the assigned significance levels, these differences were not significant (*p* = 0.02).

**Table 6:** Correlation between objective parameters and subjective/semi-objective evaluations.

**Gait (Normal Speed)**		**PD Stage**	**UPDRS I**	**UPDRS II**	**UPDRS III**	**SF-12 (MCS)**		**SF-12 (PCS)**	**Short FES-I**
Gait speed	P-value	<.0001*	<.01*	<.01*	0.02	<.01*	0.15		<.0001*
	Correlation	0.83	0.73	0.71	0.59	0.59	0.39		0.82
Stride length	P-value	<.001*	<.01*	<.01*	0.03	<.01*	0.12		<.0001*
	Correlation	0.76	0.66	0.72	0.57	0.58	0.41		0.83
Right knee angle	P-value	<.01*	<.01*	<.01*	0.01*	0.03	0.24		<.001*
	Correlation	0.72	0.71	0.75	0.62	0.32	0.32		0.81
Left knee angle	P-value	0.02	0.03	0.06	0.20	0.02	0.04		<.01*
	Correlation	0.59	0.57	0.50	0.35	0.28	0.53		0.72
Initial mid-swing	P-value	<.0001*	<0.01*	<.01*	0.01*	0.09	0.13		<.001*
	Correlation	0.84	0.71	0.70	0.62	0.45	0.41		0.80
Steady state mid-swing	P-value	<.0001*	<.01*	<.01*	0.03	0.05	0.15		<.0001*
	Correlation	0.83	0.70	0.64	0.57	0.52	0.39		0.83
**Postural Balance (eyes-open)**		**PD Stage**	**UPDRS I**	**UPDRS II**	**UPDRS III**	**SF-12 (MCS)**	**SF-12 (PCS)**		**Short FES-I**
OLΔt	P-value	0.16	<.01*	0.26	0.41	0.04	0.53		0.09
	Correlation	0.39	0.66	0.31	0.22	0.55	0.17		0.45
**iTUG**		**PD Stage**	**UPDRS I**	**UPDRS II**	**UPDRS III**	**SF-12 (PCS)**	**SF-12 (MCS)**		**Short FES-I**
Turn duration	P-value	0.04	<0.001*	<.01*	0.02	0.63	0.14		0.02
	Correlation	0.54	0.77	0.73	0.57	0.14	0.40		0.59
First turn %	P-value	0.40	<0.01*	0.10	0.15	0.53	0.02		0.36
	Correlation	0.24	0.70	0.44	0.39	0.17	0.59		0.26
Turning peak velocity	P-value	<.001*	0.05	<.01*	<.01*	0.04	0.68		<.01*
	Correlation	0.77	0.51	0.73	0.73	0.53	0.12		0.73
Turning mean velocity	P-value	<.01*	0.01*	<.01*	0.01*	0.04	0.33		<.01*
	Correlation	0.72	0.64	0.70	0.62	0.53	0.27		0.74
Sit-to-stand mean velocity	P-value	0.02	0.14	0.05	0.06	0.01*	0.81		<.01*
	Correlation	0.59	0.40	0.51	0.50	0.62	0.06		0.68
Sit-to-stand ROM	P-value	0.01*	0.27	0.03	0.04	0.02	0.91		<.01*
	Correlation	0.63	0.33	0.57	0.53	0.58	0.03		0.65
Stand-to-sit ROM	P-value	0.01*	0.05	0.06	0.69	0.14	0.69		0.02
	Correlation	0.62	0.51	0.50	0.40	0.40	0.11		0.58

Only parameters with significant Pearson correlations with subjective/semi-objective evaluations are presented (The symbol * indicates a significant correlation-0.01 was used as the significance level). Due to similarity between conditions, only normal habitual gait and the eyes-open condition for the balance test are presented. No significant correlation between SPA and subjective/semi-objective evaluations exits.

PD: Parkinson’s disease

SPA: spontaneous physical activity

iTUG: instrumented Timed-up-and-go

UPDRS: Unified Parkinson’s Disease Rating Scale

MCS: mental health composite scale

PCS: physical health composite scale

FES-I: Falls Efficacy Scale-International

ROM: range of motion

OL: open-loop.

## Discussion

### Differences between in-clinic and in-home assessment of motor impairment in PD

Both within- and between-subjects variability in motor performance measures were different between in-home and in-clinic assessments, especially in the PD group. Overall, better motor performances were observed during in-clinic assessments compared to in-home assessments for comparable tasks in both groups (Tables 4 and 5). This could be explained by the fact that in-clinic assessments were performed under supervised condition. For example, in-clinic assessment of sit-to-stand and stand-to-sit postural transitions involved TUG tests using a standardized chair. While within the in-home assessment, the duration of postural transitions was measured as the mean value of all postural transitions irrespective of the type of chair. Faster gait speed in the clinical environment may have resulted from controlling physical factors such as more uniform flooring and fewer obstacles, and/or psychological factors such as wanting to please health care providers. Specifically, large differences between in-home and in-clinic measurements in PD participants may be related to an increased awareness and motivation when performing tasks in the clinic/hospital than at home. Although the underlying mechanism is unclear, previous work demonstrated enhanced physical functioning when PD participants have more motivation and enthusiasm [33,34].

The difference between in-home and in-clinic assessments could also be explained by the fact that in-clinic assessments were performed during off-medication state, while for in-home assessment subjects might use medications. However, this may have had negligible impact on comparative observations since the in-clinic motor performance, which was during the off-medication state, were overall better than in-home assessments.

### Association between objective and subjective/semi-objective evaluations of motor impairment in PD

In gait analysis, parameters related to the speed and flexibility of movement in the lower extremities (Table 6) showed stronger correlations with PD stage compared to parameters such as gait smoothness, and asymmetric walking. This is consistent with the fact that PD patients develop rigidity with the progression of the disease, especially in the lower extremities, which can limit ankle, knee, and hip movements [35,36]. Previous studies suggested an increase in rigidity by progression of PD, especially in earlier duration of the disease (< 8 years) [37]. Since the PD group in the current study was mainly in the earlier duration of disease (< 8 years), we believe that rigidity may be the main reason for the observed reduced walking speed with the PD stage progression. The observed smaller stride length here among fallers in the PD group suggests that lower extremity rigidity may also lead to a higher risk of falling.

iTUG parameters related to speed of turning and transitions deteriorated more noticeably along with increasing disease stage (Table 6). Previous research suggested that turning abilities deteriorate faster than forward walking in early stages of PD [32,38,39]. This deterioration was observed with slower steps, a greater number of steps, and a longer delay in the last step before initiating a turn compared to healthy controls [32,38]. As the PD stage advances, asymmetric basal ganglia activity becomes more compromised; therefore, turning activity, which requires asymmetric lower limb motion, may be a better metric to demonstrate motor impairments occurring along the disease progression than forward walking [40]. Consistent with the above literatures, our results suggest that impairments in performing an asymmetric task such as turning may worsen with the PD stage. Interestingly, our results also suggest that performing an asymmetric task such as turning should produce different results between faller and non-faller PD patients.

Despite significant between-group differences in several balance and SPA parameters, no noticeable association was observed between these parameters and subjective/semi-objective evaluations (except for the OLΔt parameter that correlated with UPDRS I). This could be due to limitations of these measurement tools, the small sample size, and/or the large between-subject variability in particular for in-home measures.

The observed smaller OLΔt in the PD group compared to the controls suggests a deterioration in the balancing strategy, which may worsen with the subjective mental, behavioral, and mood characteristics of PD patients (UPDRS I). During open-loop control (short time-intervals), maintaining balance is known to be achieved by the activity of postural muscles, without using sensory feedback [41,42]. In agreement with our results, previous studies have also suggested a larger ML/AP sway rate during the open-loop control (OLslope_ML/AP_) in PD patients compared to healthy controls (Table 4) [4]. We further observed a shorter period of open-loop control strategy in the PD group, which may be attributed to higher dependency on sensory feedback (closed-loop control strategy) for maintaining balance [18,41]. Although the underlying mechanism causing the association between balancing strategy and UPDRS I needs further exploration, results from the current work suggest that postural control strategy (such as those from the open-loop/closed loop balancing mechanism) may better characterize motor impairments in the PD population, as compared to traditional body sway parameters.

Results suggest a relatively strong correlation between fear of falling as qualified by FES-I and gait related parameters quantified through both gait and iTUG tests (Table 6). In particular, with increased fear of falling a concomitant reduction in the speed and range of lower extremity motion were observed in PD group when performing in-clinic gait and iTUG tests. This may be a result of compromised postural balance during gait and iTUG, or an overall reduced physical activity with increasing fear of falling and guarding [43–45].

### Limitations and summary of findings

Limitations arise as our sample was small, and our battery of several objective assessments was challenging for individuals suffering from PD symptoms; however, we managed to recruit PD participants from all disease stages (from 1 to 4) to better associate the objective parameters with the disease severity. Additionally, the accuracy of detecting physical activity (e.g., gait speed) differs when using in-clinic compared to in-home measurement tools. For example, using the available technology, it was impossible to estimate steady state walking speed using one sensor during in-home assessments. These differences may introduce additional errors in determining the correlation between in-clinic and in-home parameters. Performing in-clinic measurements in an off-medication state may have also weaken the correlations between in-home and in-clinic measurements. The reason that in-clinic measurements were performed in an off-medication state was to reduce between-subject variability due to medication effects when correlating objective outcomes and subjective/semi-objective evaluations, as well as to resemble the common physical assessment situations that occur in labs and clinics. Finally, the focus of the current study was to explore body-worn sensor parameters with the potential to objectively characterize the physiological and biomechanical symptomology of PD. Using wearable sensor technology, we were able to perform all in-clinic objective assessments in a shorter timeframe (between 30 to 45 minutes) to avoid participant exhaustion. A large number of objective parameters were considered within the current study; however, several additional parameters exist which could be explored in future studies to more accurately characterize motor impairment in those with PD.

In summary, detecting motor impairments was more difficult when measuring physical activities during natural daily activity as compared to objective tests within the lab or clinic environment in those with PD, unlike in healthy controls. Furthermore, parameters related to speed and range of movement, especially during an asymmetrical task such as turning, may be more sensitive to PD stage when compared to healthy controls and could prove promising as indicators of change over time (e.g., with changes in disease progression, medication management, or other interventions).

### Clinical implications and future directions

Based on the compiled results extracted from the current study, we made the following recommendations regarding objective assessment of motor performance in Parkinson’s patients:

1. Overall, detecting motor impairments is harder using SPA monitoring compared to in-clinic measurements, likely due to a higher between-subject variability in SPA outcome measures. Therefore, a larger sample size is required when studying SPA in PD patients in-home compared to in-clinic assessments.
2. To better assess physical changes due to the progression of either disease or treatment, parameters related to speed and flexibility of movements (such as walking speed, range of lower extremity motion) and asymmetric tasks (such as turning) are preferred.
3. Overall, in-clinic gait and iTUG tasks that involve more rapid movements compared to balance and SPA are better options for detecting differences in motor impairments at different stages of PD.
